# Editorial: Plant signaling in response to environmental stresses

**DOI:** 10.3389/fpls.2023.1282465

**Published:** 2023-09-21

**Authors:** Van Hien La, Tae-Hwan Kim, Alejandro Calderon-Urrea, Tibor Janda

**Affiliations:** ^1^ Center of Crop Research for Adaptation to Climate Change, Thai Nguyen University of Agriculture and Forestry, Quyet Thang, Thai Nguyen, Vietnam; ^2^ Department of Animal Science, Institute of Agricultural Science and Technology, College of Agriculture & Life Science, Chonnam National University, Gwangju, Republic of Korea; ^3^ Department of Biology, California State University, Fresno, Fresno, CA, United States; ^4^ Department of Plant Physiology and Metabolomics, Agricultural Institute, Center for Agriculture Research, Eötvös Loránd Research Network (ELKH), Martonvásár, Hungary

**Keywords:** abiotic stress, environment stress, hormone signaling, ROS signaling, phenotypic plasticity

In nature, plants constantly interact with both abiotic and biotic environments. Recent studies have often converged on plant-stress interactions by modulating and eliciting precise signals. Advances in molecular techniques and mining of big datasets have increased our understanding of these processes, revealed new levels of complexity, and opened new research directions. This Research Topic aims to provide an interdisciplinary understanding of how plants use physiological, biochemical, and molecular genetic mechanisms to adapt to adverse environments. The contributions included in this Research Topic provide new insights into the responses and adaptations of various crops to abiotic stresses.

In a study of this Research Topic, Jin et al. indicated that fluctuating desert environments induce temporal variation in the photosystem II (PSII)-energy partitioning response. The authors revealed that different responses to PSII-energy allocation were influenced by photosynthetically active radiation (*PAR*), air temperature (*T_a_*), and vapor pressure deficit (*VPD*) at a diurnal scale. In contrast, PSII-energy partitioning on a seasonal scale displayed greater variability among the different environmental variables, such as photochemical efficiency (*Φ*_PSII_) and non-regulatory thermal dissipation (*Φ*_NPQ_, *Φ*_NO_), being more predisposed to changes in *T_a_*, and *Φ*_NPQ_ to changes in *VPD*, acclimatize to excessive *PAR*, dry-air conditions, and prolonged drought. Photosynthesis in plants is particularly susceptible to environmental fluctuations. Similarly, plant photosynthetic activity and growth are related to light intensity. Low light intensity caused by shading significantly reduces plant growth and biomass, probably because of the reduced photosynthetic rate (Wang et al., 2012). Interestingly, Zhang et al. addressed clonal integration (*Glechoma longituba*), and high-nutrient supplements not only significantly increased the growth of apical portions, but also enhanced plant growth and biomass under shaded light conditions. Collectively, these studies revealed that the pictures emerged from environmentally induced variations in photosynthetic processes as a function of plant adaptation.

Plants perceive stress signals through internal receptors, such as G-protein, kinase, reactive oxygen species (ROS), and calcium, which trigger molecular cascades to transmit signals (Devireddy et al., 2021). Among these, calcium-permeable channels in the plasma membrane play a vital role in plant response to environmental stress. Silamparasan et al. addressed the significance of calcium-dependent protein kinase (CDPKs)-mediated phosphorylation of serine (Ser)-856 of glutamate receptor-like (GLR)3.6 protein, which plays an essential role in salt and abscisic acid (ABA) response in Arabidopsis by modulating Ca^2+^ signaling. Additionally, Ca^2+^-mediated CDPK16 phosphorylates GLR3.6, which regulates root growth under normal and salt-stress conditions. On the other hand, in guard cells, H_2_O_2_ triggers an influx of cytosolic calcium (Ca^2+^) to regulate ABA-induced stomatal closure in Arabidopsis plants (Pei et al., 2000). Evidence has demonstrated that H_2_O_2_-induced Ca^2+^ ion flux is involved in H_2_O_2_ perception and signaling pathways (Demidchik and Shabala, 2018). Additionally, Available at: Wu et al. (2020) identified hydrogen peroxide-induced Ca^2+^ increase 1 (HPCA1) as a leucine-rich repeat receptor kinase induced by H_2_O_2_. HPCA1 mediates H_2_O_2_-induced activation of Ca^2+^ channel signals in guard cells and is required for stomatal closure. Therefore, HPCA1 is involved in the perception of extracellular H_2_O_2_ in response to various external stressors and internal cues in plants.

Recently, abscisic acid application has been shown to partially improve water use efficiency (WEU). Roeder et al. found that ABA-related cyano cyclopropyl compounds (CCPs) play a pivotal role in minimizing leaf transpiration. Several CCPs activate ABA signaling, such as CCP1, CCP2, and CCP5, which are an order of magnitude more efficient than ABA in minimizing transpiration in Arabidopsis plants. Among these, CCP2 mediated an increase in water use efficiency superior to ABA, without trade-offs in biomass accumulation in a progressive drought experiment. Thus, ABA and other chemically stable ABA agonists have the potential to improve crop water productivity. Furthermore, ABA is known to be a phytohormone responsible for stomatal closure, and ABA receptors including the REGULATORY COMPONENT OF ABA RECEPTOR (RCAR) and PYRABACTIN RESISTANCE 1-LIKE (PYL) (PYR/PYL/RCAR) play a central role in executing ABA’s role in water relations (Cutler et al., 2010; Vaidya et al., 2019). Among these ABA receptors, RCAR and PYL are the most targeted for ABA sensitivity and water productivity. Overexpression of ABA receptors *RCAR6/PYL12* increased water use efficiency (WUE) by up to 40% in Arabidopsis (Yang et al., 2016). In addition, overexpression of *TaPYL1/2/4/6* in wheat increases ABA sensitivity and significantly lowers a plant’s lifetime water consumption (Mega et al., 2019). Physiological analyses of *TaPYL4* overexpressing plants showed that the water-saving trait is a consequence of reduced transpiration during water deficits (Mega et al., 2019).

Furthermore, one contribution pertains to transcriptome analysis aimed at gaining insight into the molecular response of soybean drought response to drought mepiquat chloride pretreatment. Wang et al. identified DEGs in drought-tolerant and drought-sensitive soybean genotypes and identified candidate genes such as LOC100816177, SOMT-2, LOC100784120, LOC100797504, LOC100794610, and LOC100819853, which are crucial for the drought resistance of soybeans. Taken together, this study indicated that 2-oxocarboxylic acid metabolism and isoflavone biosynthetic pathways are the core pathways by which mepiquat chloride regulates soybean drought response.

In summary, the five articles published on the Research Topic provide illustrative examples of the research area of plant signaling responses to environmental stress by highlighting the complexity of the connections between physiological, key signaling, and metabolic pathways in plants. Thus, we hope that these compiled articles provide new insights into this topic and expand the scope of future research.

## Author contributions

VHL: Writing – orginal draft & editing. T-HK: Writing – review & editing. AC-U: Writing – review & editing. TJ: Writing – review & editing.
